# Mild stress accumulation limits GABAergic synaptic plasticity in the lateral habenula

**DOI:** 10.1111/ejn.15581

**Published:** 2022-01-03

**Authors:** Arnaud L. Lalive, Alvaro Nuno‐Perez, Anna Tchenio, Manuel Mameli

**Affiliations:** ^1^ Department of Fundamental Neuroscience University of Lausanne Lausanne Switzerland; ^2^ Institut du Fer à Moulin, Inserm UMR‐S 839 Paris France

**Keywords:** inhibitory transmission, long‐term depression, nitric oxide, presynaptic release, stress

## Abstract

Animals can cope with isolated stressful situations without enduring long‐term consequences. However, when exposure to stressors becomes recurrent, behavioural symptoms of anxiety and depression can emerge. Yet, the neuronal mechanisms governing responsivity to isolated stressor remain elusive. Here, we investigate synaptic adaptations following mild stress in the lateral habenula (LHb), a structure engaged in aversion encoding and dysfunctional in depression. We describe that neuronal depolarization in the LHb drives long‐term depression of inhibitory, but not excitatory, synaptic transmission (GABA LTD). This plasticity requires nitric oxide and presynaptic GABA_B_ receptors, leading to a decrease in probability of GABA release. Mild stressors such as brief social isolation, or exposure to novel environment in the company of littermates, do not alter GABA LTD. In contrast, GABA LTD is absent after mice experience a novel environment in social isolation. Altogether, our results suggest that LHb GABAergic plasticity is sensitive to stress accumulation, which could represent a threshold mechanism for long‐term alterations of LHb function.

## Graphical abstract

Neuronal depolarization induces long‐term depression of GABA (GABA LTD), but not glutamate transmission in the LHb. GABA LTD is induced postsynaptically and expressed presynaptically via nitric oxide as a retrograde messenger and GABA_B_ receptors. Exposure to single mild stressors (novelty or social isolation) does not affect GABA LTD, but their accumulation prevents the expression of the plasticity.

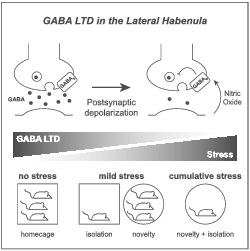

**Abbreviations**
ACSF: artificial cerebrospinal fluid
EPSC: excitatory postsynaptic current
IPSC: inhibitory postsynaptic current
GABA: gamma‐aminobutyric acid
GIRK: G protein‐coupled inwardly‐rectifying potassium channel
LHb: lateral habenula
LTD: long‐term depression
NO: nitric oxide
NOS: nitric oxide synthase
sGC: soluble guanylyl cyclase

## 1 INTRODUCTION

Stress is the physiological reaction to a situation perceived as demanding or threatening. Isolated or mild stressors induce physical and emotional tension that rapidly dissipates after individuals effectively deal with the source of stress (Willner et al., 2013). If exposure to stressors increases in intensity or recurrence, the subsequent cumulative stress response can induce long‐lasting aversive states of emotional distress and mental disorders, like anxiety and depression (Slattery & Cryan, 2017). Despite high incidence of stress‐related diseases across societies, the cellular mechanisms responsible for stress susceptibility, accumulation and the transition to pathological states remain limited.

Stressful experiences engage multiple brain circuits, including the lateral habenula (LHb), an evolutionary conserved epithalamic structure engaged in aversive encoding (Hu et al., 2020). LHb neurons increase their activity in response to a variety of aversive stimuli, or stressors, ranging from mild to severe intensity. In turn, LHb excitation shapes behavioural strategies to either avoid or escape stressful situations (Andalman et al., 2019; Grillner et al., 2018; Lawson et al., 2014; Lecca et al., 2017, 2020; Matsumoto & Hikosaka, 2007; Trusel et al., 2019; Wang et al., 2017). Importantly, LHb neurons become hyperactive in both depressed humans and animal models of depression, and normalizing LHb activity with pharmacological or electrical approaches rescues behavioural symptoms (Li et al., 2011; Sartorius et al., 2010; Tchenio et al., 2017; Yang et al., 2018). Thus, the LHb is a stress‐responsive structure essential for the onset and treatment of depression.

In rodents, protocols of exposure to high‐intensity stressors (i.e. electric shocks) have been used to model depression and identify adaptations in LHb synaptic transmission and neuronal activity. As early as 1 h after a high stress experience, GABA_B_ receptor internalization weakens neuronal inhibition, leading to membrane hyperexcitability and exacerbated burst firing in LHb neurons (Lecca et al., 2016; Tchenio et al., 2017; Yang et al., 2018). Furthermore, high stress protocols also alter fast neurotransmission. Indeed, protracted foot‐shock experience modifies excitatory glutamatergic tone (Li et al., 2011; Nuno‐Perez et al., 2021) and reduces GABAergic function onto LHb neurons (Shabel et al., 2014). Importantly, these alterations contribute causally to depressive‐like symptoms. Overall, these results support that neuronal adaptations in the LHb represent a hallmark of stress‐driven depression (Hu et al., 2020).

In contrast to high‐intensity stressors, acute exposure to mild stressors does not induce depressive‐like phenotypes while readily increasing the activity of LHb neurons (Matsumoto & Hikosaka, 2007; Wang et al., 2017). Instead, chronic exposure to mild stressors is necessary to drive the emergence of aversive states and adaptations in the LHb in a fraction of susceptible animals, resembling depression in humans (Cerniauskas et al., 2019; Willner, 2017). These results suggest that LHb neurons can monitor cumulative exposure to mild stressors, which may or may not culminate in depression‐related alterations. Such a surveillance system may rely on the well‐established stressor‐driven LHb excitation. Yet, whether and how neuronal excitation or mild stressors induce plastic adaptations in the LHb remains elusive.

To answer these questions, we used slice electrophysiology to first test how excitation of LHb neurons, mimicking in vivo response to aversive stimuli, affects synaptic transmission. We found that neuronal depolarization induces a long‐term depression of inhibitory transmission (GABA LTD), but not excitation. This form of plasticity relies on a reduction in presynaptic GABA release, a mechanism related to depressive states (Shabel et al., 2014). Finally, we exposed mice to mild stressors such as novelty exploration and brief social isolation, which do not induce depressive‐like phenotypes. We observed that only the cumulative experience of these stressors impaired GABA LTD. Thus, our results highlight that GABA plasticity is a phenomenon sensitive to physiological stress thresholds and suggest a role of the LHb in stress response before the emergence of pathological traits.

## 2 METHODS

### 2.1 Animals

All procedures were done in accordance with the veterinary office of Vaud (Switzerland, license VD3171.1). Animals (C57BL6/6JR, Janvier, 8‐ to 20‐week‐old male mice) were maintained on a 12/12 h light/dark cycle and fed ad libitum. Behavioural experiments occurred during the light cycle.

### 2.2 Slice electrophysiology

The mice were anaesthetized (ketamine/xylazine; 150 mg/100 mg kg^−1^), sacrificed, and their brains were transferred in ice‐cold carbogenated (95% O_2_/5% CO_2_) solution, containing (in mM) choline chloride 110; glucose 25; NaHCO_3_ 25; MgCl_2_ 7; ascorbic acid 11.6; sodium pyruvate 3.1; KCl 2.5; NaH_2_PO_4_ 1.25; CaCl_2_ 0.5. Coronal brain slices (250 μm thickness) were prepared and transferred for 5 min to warmed solution (34°C) of identical composition, before transfer at room temperature in carbogenated artificial cerebrospinal fluid (ACSF) containing (in mM) NaCl 124; NaHCO_3_ 26.2; glucose 11; KCl 2.5; CaCl_2_ 2.5; MgCl_2_ 1.3; NaH_2_PO_4_ 1. During recordings, slices were immersed in ACSF and continuously superfused at a flow rate of 2.5 ml min^−1^ at 32°C, except for the cPTIO recordings and matching controls, which were performed at 25°C. Neurons were patch‐clamped using borosilicate glass pipettes (3–4 MΩ; Phymep, France) under an Olympus‐BX51 microscope (Olympus, France). Signal was amplified, filtered at 5 kHz and digitized at 10 kHz (MultiClamp 200B; Molecular Devices, USA). Data were acquired using Igor Pro with NIDAQ tools (WaveMetrics, USA). Access resistance was continuously monitored with a −4 mV step delivered at 0.1 Hz. Extracellular stimulation from AMPI ISO‐Flex stimulator controlled by a Master‐8 (AMPI, Israel) was delivered through a monopolar electrode inside an ACSF‐filled borosilicate pipette placed in the LHb. All recordings were made in voltage‐clamp configuration, and neurons were held at ‐50 mV with an internal solution containing (in mM): CsCl 130; NaCl 4; creatine phosphate 5; MgCl_2_ 2; Na_2_ATP 2; Na_3_GTP 0.6; EGTA 1.1; HEPES 5, QX‐314 5. IPSCs were recorded with APV (100 μM, NMDAR antagonist) and NBQX (10 μM, AMPAR antagonist) in the ACSF, whereas EPSCs were recorded in the presence of picrotoxin (100 μM, GABA_A_R antagonist). All drugs were purchased from Hello Bio (UK).

### 2.3 Behaviour

The familiar environment consisted of the homecage in which mice were housed in groups of three to five littermates for at least 1 week after arrival from the Janvier facility. The novel environment consisted of a 15 × 20 cm chamber with a metal grid floor in a dark, soundproof compartment (Ugo Basile, Italy). Exposure to novelty consisted of 20‐min sessions once a day for 3 days, either in the company of littermates (novelty mild stress condition) or alone (stress accumulation condition). For the social isolation mild stress condition, littermates were removed from the homecage to leave a single animal alone in its familiar environment for 3 days before recordings.

### 2.4 Quantification and statistical analysis

Online and offline analyses of evoked synaptic currents were performed using Igor Pro 6 (WaveMetrics, USA). Spontaneous postsynaptic currents recordings were manually analyzed offline using MiniAnalysis (Synaptosoft Inc, USA). Sample size was predetermined on the basis of published studies and in‐house expertise. Animals were randomly assigned to experimental groups. All data are represented as mean ± SEM, and individual data points are shown. Data collection and analysis were not performed blinded to experimental conditions. LTD significance was assessed statistically by analyzing the last 5 min of recordings compared with baseline. Statistical comparisons were done in Prism (GraphPad) with the following methods: Wilcoxon matched pairs signed rank test, one‐sample *t*‐test, repeated measures one‐way ANOVA followed by Tukey's multiple comparison test. Significance was denoted as ***p < 0.001, **p < 0.01 and *p < 0.05.

## 3 RESULTS

### 3.1 Depolarization‐induced GABA LTD onto LHb neurons

**FIGURE 1:**
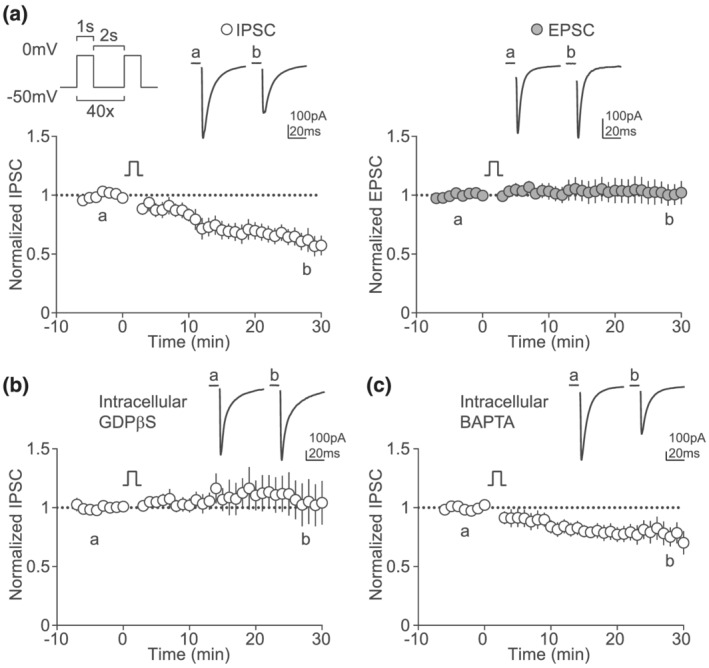
Postsynaptic depolarization drives LTD of GABAergic transmission in LHb neurons. (a) Depolarization protocol and example IPSC traces before and 30 minutes after induction (top). Time course of normalized IPSC amplitude (bottom, *n* = 12, Wilcoxon, *p* = 0.0024). (b) Example traces and time course of normalized EPSC amplitude before and after the depolarization protocol (*n* = 8, Wilcoxon, *p* = 0.3828). (c) Example traces and time course of normalized IPSC amplitude before and after the depolarization protocol with GDPβS (1 mM) added to the intracellular recording solution (*n* = 12, Wilcoxon, *p* = 0.4238). (d) Example traces and time course of normalized IPSC amplitude before and after the depolarization protocol with BAPTA (10 μM) added to the intracellular recording solution (*n* = 10, Wilcoxon, *p* = 0.0195)

Neuronal depolarization is a key event for the induction of synaptic plasticity in the brain. Because aversive stimuli depolarize and increase firing of LHb neurons, we investigated whether depolarization affects synaptic transmission in the LHb. To do so, we performed whole‐cell patch clamp recordings from LHb neurons in acute brain slices of mice. We evoked excitatory and inhibitory postsynaptic currents (EPSC, IPSC) with an electrode placed within the LHb. We found that repeated postsynaptic depolarization induced long‐term depression of GABA_A_ receptor‐mediated IPSC (GABA LTD; Figure 1a). In contrast, this protocol did not affect the amplitude of AMPA receptor‐mediated EPSC (Figure 1a). GABA LTD was induced in the presence of AMPA and NMDA receptor antagonists, excluding the involvement of major excitatory ionotropic receptors in this form of plasticity. Thus, we tested whether metabotropic receptors and G protein signalling participate to the induction of GABA LTD. To do so, we included GDPβS in the intracellular recording solution. This manipulation blocked GABA LTD (Figure 1b). As both depolarization and G protein activation can lead to a rise in postsynaptic calcium, we assessed if calcium was necessary for GABA LTD. Intracellular BAPTA, a calcium chelator, did not affect GABA LTD (Figure 1c). Altogether, these results suggest that LHb depolarization‐driven GABA LTD is induced postsynaptically, requires G protein signaling and is independent of calcium.

### 3.2 Nitric oxide release underlies LHb GABA LTD

**FIGURE 2:**
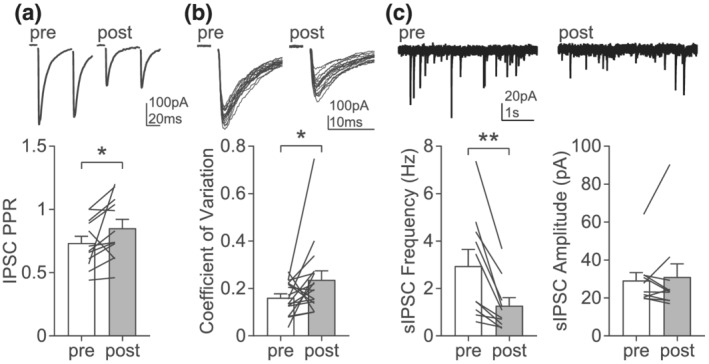
GABA LTD is expressed presynaptically. (a) Example traces (top) and average IPSC paired‐pulse ratio (PPR, bottom) before (pre) and after (post) the depolarization protocol (*n* = 12, Wilcoxon, *p* = 0.0342). (b) Example traces of IPSC amplitude variability (top) and average coefficient of variation (bottom) before and after the depolarization protocol (*n* = 17, Wilcoxon, *p* = 0.0395). (c) Example traces of spontaneous IPSC (sIPSC) before and after the depolarization protocol and average frequency (*n* = 10, Wilcoxon, *p* = 0.002) and amplitude (*n* = 10, Wilcoxon, *p* = 0.2783)

We then sought to identify the expression site of GABA LTD. Depolarization induced a significant increase in paired‐pulse ratio, simultaneous to the depression of IPSC amplitude (Figure 2a), suggesting a reduction in probability of release. Accordingly, we also observed an increase in the coefficient of variation of IPSC amplitude after LTD induction (Figure 2b; Faber & Korn, 1991). Finally, we found a significant decrease in the frequency, but not amplitude of spontaneous IPSCs after depolarization, compared with baseline values (Figure 2c). These observations highlight a consistent decrease in probability of release, supporting presynaptic expression of GABA LTD.

**FIGURE 3:**
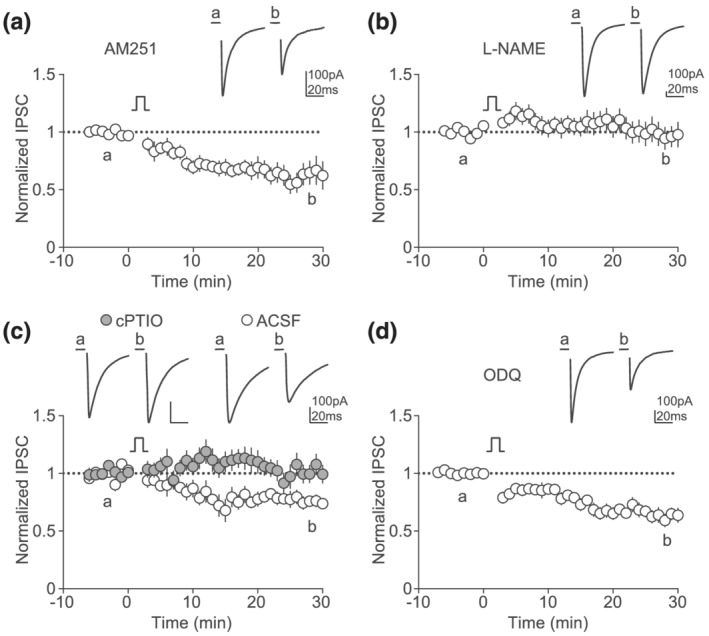
GABA LTD is mediated by nitric oxide. (a) Example traces and time course of normalized IPSC amplitude before and after the depolarization protocol with AM251 (2 μM) present in the extracellular recording solution (*n* = 7, Wilcoxon, *p* = 0.0313). (b) Example traces and time course of normalized IPSC amplitude before and after the depolarization protocol with L‐NAME (200 μM) present in the extracellular recording solution (*n* = 11, Wilcoxon, *p* = 0.6221). (c) Example traces and time course of normalized IPSC amplitude before and after the depolarization protocol with cPTIO (30 μM, grey circles, *n* = 11) added to the extracellular recording solution, or without (open circles, *n* = 9, t‐test, cPTIO vs. ACSF p = 0.022). (d) Example traces and time course of normalized IPSC amplitude before and after the depolarization protocol with ODQ (10–100 μM) present in the extracellular recording solution (*n* = 14, Wilcoxon, *p* = 0.0001)

Because postsynaptic depolarization drives presynaptic changes in probability of release at GABAergic terminals, we hypothesized that GABA LTD expression requires the release of a retrograde messenger, as it occurs in many brain structures (Chevaleyre & Castillo, 2003; Nugent et al., 2007; Wamsteeker Cusulin et al., 2013). Neuronal depolarization can engage, among others, the release of endocannabinoids or the synthesis of nitric oxide (NO), a gas that diffuses freely across membranes and differentially regulates synaptic transmission depending on neurotransmitter, cell type and brain region (Chu et al., 2015; Lev‐Ram et al., 1995; Nugent et al., 2007; Shibuki & Okada, 1991). Thus, we tested whether endocannabinoids or NO contribute to GABA LTD. Blocking CB1 receptors, the major presynaptic target of endocannabinoids, did not prevent GABA LTD (Figure 3a). In contrast, incubation of slices with L‐NAME, an inhibitor of nitric oxide synthase (NOS), blocked the depolarization‐induced decrease in IPSC amplitude (Figure 3b). Furthermore, GABA LTD was absent after bath application of an NO scavenger (cPTIO; Figure 3c). Together, these results indicate a role of NO in retrograde signalling in the LHb. Soluble guanylyl cyclase (sGC) is the main known target of NO. Intriguingly, blocking sGC activation with ODQ did not affect GABA LTD, suggesting that NO targets a different substrate (Figure 3d).

### 3.3 Nitric oxide and G_i/o_ signalling crosstalk for GABA LTD

**FIGURE 4:**
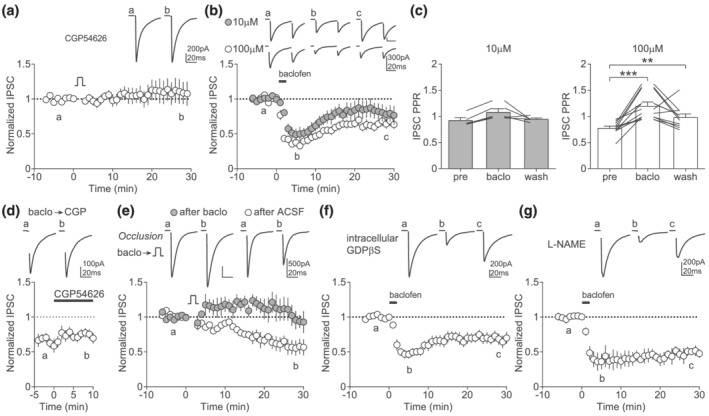
GABA LTD requires presynaptic GABA_B_ receptors. (a) Example traces and time course of normalized IPSC amplitude before and after the depolarization protocol with CGP54626 (2 μM) present in the extracellular recording solution (*n* = 11, Wilcoxon, *p* = 0.9463). (b) Example traces and time course of normalized IPSC amplitude before and after a 2‐min bath application of baclofen (10 μM, grey circles, *n* = 5, Wilcoxon, *p* = 0.1875; 100 μM, open circles, *n* = 10, Wilcoxon, *p* = 0.002). (c) Average IPSC PPR before (pre), 5 min (baclo) and 30 min (wash) after baclofen application (left 10 μM, one‐way ANOVA RM, *F*(1.062,4.247) = 5.071, *p* = 0.0829; right 100 μM, one‐way ANOVA RM, *F*(1.627,16.27) = 16.63, *p* = 0.0002, post hoc Tukey's, pre vs. baclo *p* = 0.0006, pre vs. wash *p* = 0.0094). (d) Example traces and time course of normalized IPSC amplitude where bath application of CGP54626 (2 μM) occurs 30 min following brief 100‐μM baclofen application (*n* = 7, Wilcoxon, a vs. b *p* = 0.2969). (e) Occlusion experiment where the depolarization protocol was applied 30 min after brief bath application of 100 μM baclofen (grey circles, *n* = 6) or control ACSF (open circles, *n* = 5, t‐test, ACSF vs. baclo *p* = 0.0325). Top, example traces; bottom, time course of normalized IPSC amplitude.(f) Example traces and time course of normalized IPSC amplitude before and after a 2‐min bath application of baclofen (100 μM) with GDPβS (1 mM) added to the intracellular recording solution (*n* = 10, Wilcoxon, *p* = 0.0059). (g) Example traces and time course of normalized IPSC amplitude before and after a 2‐min bath application of baclofen (100 μM) with L‐NAME (200 μM) present in the extracellular recording solution (*n* = 10, one‐sample *t*‐test, *p* = 0.0013)

Next, we investigated how NO could decrease the probability of GABA release. Presynaptic G_i/o_ protein signalling represents a most efficient mechanism to weaken synaptic transmission, notably through GABA_B_ receptor activation (Lüscher et al., 1997). Thus, we designed a series of experiments to identify whether presynaptic GABA_B_ receptors and G_i/o_ signalling would contribute to GABA LTD. First, we bath applied CGP54626, a GABA_B_ receptor antagonist throughout the recording. This manipulation blocked GABA LTD, suggesting the involvement of GABA_B_ receptors (Figure 4a). Then, we asked whether activation of GABA_B_ receptor would be sufficient to mimic GABA LTD. Brief application of the GABA_B_ receptor agonist baclofen rapidly reduced IPSC amplitude, an effect that partially washed out 30 min after application (Figure 4b). This effect was dose dependent, with a saturating concentration (100 μM) leading to stable LTD, along with a significant increase in paired‐pulse ratio (Figure 4c). This long‐lasting effect was not due to incomplete baclofen washout or sustained GABA_B_ receptor activation, as bath application of a GABA_B_ receptor antagonist did not reverse the baclofen LTD (Figure 4d). Thus, baclofen drives a long‐lasting decrease in presynaptic GABA release that resembles GABA LTD. If depolarization‐induced NO engages presynaptic G_i/o_ signalling, then GABA_B_ receptor activation should interfere with GABA LTD. Thus, we applied baclofen for 2 min, waited 20 min until baclofen‐LTD was established (Figure 4b), to finally record baseline IPSCs and launch the depolarization protocol. In these conditions, we did not observe any change in IPSC amplitude, although GABA LTD was present in control conditions (Figure 4e). These results support that presynaptic GABA_B_ receptor activation occludes GABA LTD, suggesting shared signalling pathways. Importantly, intracellular GDPβS, which blocks both postsynaptic G protein signalling and depolarization‐induced GABA LTD (Figure 1b), did not prevent baclofen LTD (Figure 4f). This observation supports the involvement of presynaptic, but not postsynaptic, GABA_B_ receptors in baclofen LTD. Furthermore, NOS inhibition had no effect on baclofen LTD (Figure 4g), suggesting that GABA_B_ receptor/G_i/o_ activation occurs downstream of NO synthesis. Yet, whether postsynaptic GABA_B_ receptors contribute to a discrete phase of the plasticity cannot be ruled out. Altogether, these results suggest that NO engages presynaptic GABA_B_ receptors or G_i/o_ signalling to drive a long‐lasting decrease in neurotransmitter release at GABAergic inputs to LHb.

### 3.4 GABA LTD as a substrate for stress thresholds

**FIGURE 5:**
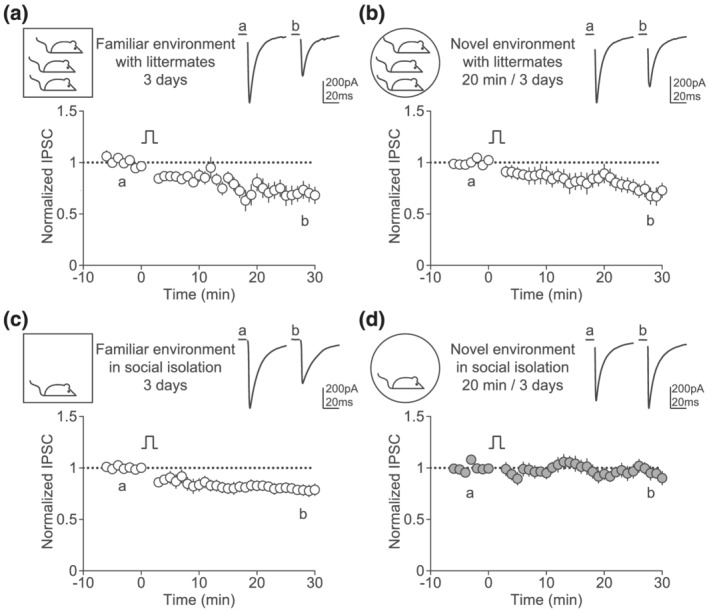
Cumulative stress disrupts GABA LTD. (a) Top left, cartoon showing no stress condition where mice are kept in a familiar environment with littermates. Top right and bottom, example traces and time course of normalized IPSC amplitude before and after the depolarization protocol (*n* = 8, Wilcoxon, *p* = 0.0156). (b) Top left, cartoon showing mild stress condition where mice are exposed to a novel environment 20 min per day for 3 days, with littermates. Top right and bottom, example traces and time course of normalized IPSC amplitude before and after the depolarization protocol (*n* = 10, Wilcoxon, *p* = 0.002). (c) Top left, cartoon showing mild stress condition where mice are isolated in their familiar homecage. Top right and bottom, example traces and time course of normalized IPSC amplitude before and after the depolarization protocol (*n* = 12, Wilcoxon, *p* = 0.0015). (d) Top left, cartoon showing cumulative stress condition where mice are exposed to a novel environment 20 min per day for 3 days, in social isolation. Top right and bottom, example traces and time course of normalized IPSC amplitude before and after the depolarization protocol (*n* = 11, Wilcoxon, *p* = 0.4648)

Protracted and harsh stress exposure affects synaptic transmission and plasticity in the LHb (Li et al., 2011; Nuno‐Perez et al., 2021; Shabel et al., 2014; Yang et al., 2018). In turn, these adaptations are central to the emergence of anxiety traits and depressive‐like behavioural symptoms (Hu et al., 2020). However, whether LHb synaptic transmission becomes aberrant when animals cope with mildly aversive stimuli remains unknown. Therefore, we asked whether experiencing mild stress would alter GABA LTD in order to demonstrate and understand the physiological relevance of this plasticity. We focused on transient social isolation and exposure to novel environment, which are considered as mild stressors in animals and humans (Willner, 2017). As previously established, animals housed in groups in their familiar environment showed reliable GABA LTD (Figure 5a). Exposing mice in groups of littermates to a novel environment (20 min per day, for 3 days) did not alter GABA LTD (Figure 5b). Similarly, we were able to induce the plasticity in mice socially isolated in their familiar environment (homecage; Figure 5c). However, GABA LTD was absent after mice were exposed to the novel environment in social isolation (Figure 5d). Thus, the accumulation of stressors engages synaptic adaptations in the LHb that affect GABA LTD.

## 4 DISCUSSION

We used postsynaptic depolarization in LHb neurons to mimic in vivo increases in activity following aversive stimuli, which is also a standard model for the study of synaptic plasticity in acute brain slices (Castillo et al., 2011; Malenka & Bear, 2004). We describe a form of GABA LTD that is induced postsynaptically and expressed presynaptically, via NO and presynaptic GABA_B_ receptors. Notably, this form of plasticity has a behavioural relevance, as it is absent when mice experience mild stress accumulation.

### 4.1 Mechanistic understanding of GABA LTD

We found that depolarization of LHb neurons led to long‐term decrease in probability of release at GABAergic terminals. Our results suggest that NO acts as the retrograde messenger, linking postsynaptic induction to presynaptic expression. In terms of induction, a current model of postsynaptic NOS activation involves glutamate release, NMDA receptor activation and intracellular calcium (Chu et al., 2015; Lange et al., 2012; Nugent et al., 2007). In the LHb, we found that postsynaptic depolarization drives NO synthesis, independently of NMDA receptors or increases in calcium. In the thalamus, postsynaptic bursting alone also activates NOS, yet in a calcium‐dependent manner (Sieber et al., 2013). LHb neurons may diverge in this regard, with the expression of the inducible form of NOS, whose activation does not require calcium to generate NO (Béchade et al., 2014; Ruan et al., 1996). In addition, we show that postsynaptic G protein signalling is necessary, although the identity of the G protein coupled receptor remains elusive. We highlight the importance of presynaptic GABA_B_ receptors for GABA LTD expression (see below). Yet, we cannot fully discard a potential involvement of postsynaptic GABA_B_ receptors for LTD induction, even if G_i/o_ seems to limit NO metabolites (Ishizuka et al., 2000). Alternatively, metabotropic glutamate receptors (mGluR) are potent modulators of synaptic transmission. mGluR1 activation in the LHb also induces an LTD of inhibitory transmission. However, the expression is postsynaptic and involves GABA_A_ receptor trafficking (Valentinova & Mameli, 2016), suggesting that mGluR1 are not involved in NO‐dependent, presynaptic GABA LTD. The role of other metabotropic receptors remains to be tested.

Regarding the GABA LTD expression mechanism, sGC is the main known target of NO in synaptic terminals. Yet, we observed that inhibiting sGC did not block GABA LTD, suggesting that NO may engage unusual presynaptic partners. This hypothesis is consistent with the fact that we observe NO‐dependent GABA LTD, though previous studies reported NO/sGC‐dependent GABA potentiation (Chu et al., 2015; Nugent et al., 2007; Sieber et al., 2013). Instead, we show that antagonism of GABA_B_ receptors blocks GABA LTD. Furthermore, transient activation of presynaptic G_i/o_ pathway through GABA_B_ receptor agonist baclofen fully mimics and occludes GABA LTD. Altogether, these results suggest shared signalling pathways between NO and GABA_B_/G_i/o_ signalling. It remains unclear whether NO could directly interact with GABA_B_ receptor or with specific elements of the downstream molecular pathway.

Our mechanistic dissection of GABA LTD in the LHb highlights the variety of molecular pathways leading to NOS activation and, most importantly, suggest that non‐canonical, unidentified targets of NO determine the direction of NO's influence on synaptic transmission. Strikingly, neuronal depolarization had no effect on excitatory transmission in the LHb, where presynaptic LTD seems to be mediated rather by endocannabinoids (Park et al., 2017; Valentinova & Mameli, 2016). This discrepancy suggests that LHb neurons possess the machinery to release various retrograde messengers, whereas their molecular targets differ at excitatory and inhibitory inputs and orchestrate synaptic plasticity.

### 4.2 Relating depolarization‐driven GABA LTD to aversion‐induced LHb hyperactivity

Various stimuli activate LHb neurons, ranging from mildly aversive air puffs to harsher stressors like painful electrical shocks. We show that membrane depolarization of LHb neurons depresses GABAergic transmission within minutes, suggesting that exposure to stressors in vivo may induce similar plasticity. This is consistent with the fact that depolarization did not induce any further decrease of GABAergic transmission after exposure to stressors, supporting an occlusion of GABA LTD. This interpretation implies that in vivo experience of stress induces a long‐lasting plasticity still present 24 h later, which we have not shown directly. The involvement of NO in GABA LTD is quite fitting, as exposure to stressful stimuli is associated with enhanced NOS activation (Kumar & Chanana, 2017). Yet, we cannot fully discard that the absence of GABA LTD results from an alteration of the molecular pathways involved in plasticity induction or expression. Further investigation should manipulate the GABA LTD machinery in vivo to identify how experience affects the plasticity of inhibitory transmission in the LHb. Knowledge related to inhibitory innervation into the LHb and the molecular diversity of receptors subtypes represent a starting point to specifically target GABAergic function in LHb (Geisler et al., 2003; Wagner et al., 2016). Indeed, losing the ability to modulate GABAergic transmission onto LHb neurons is likely to have functional consequences. For example, mGluR‐dependent LTD of GABA_A_ receptors directly influences the transformation of excitatory inputs into action potentials in LHb neurons (Valentinova & Mameli, 2016). Similarly, aversive experience involving high stress levels downregulates GABA_B_–GIRK expression, resulting in neuronal hyperexcitability (Lecca et al., 2016). Thus, inhibitory strength tightly modulates the neuronal output of LHb neurons, and GABA LTD may change LHb responsivity to subsequent exposure to stressful stimuli. Accordingly, stress‐dependent modulation of GABA LTD may occur at inputs carrying stress‐related information, such as the entopeduncular nucleus (Shabel et al., 2014). Future studies should address the input‐specific role and plasticity of inhibition in stress.

### 4.3 Synaptic substrate of stress thresholds

Plastic adaptations in the LHb are central to the emergence of stress‐related diseases such as anxiety and depression. Yet, humans and animals regularly face stressful situations, cope perfectly with them and move on unscathed. We described that a mildly stressful experience—exploration of a novel environment or brief social isolation—does not affect GABA LTD in the LHb. However, the combination of these stressors is necessary to affect GABA LTD. Thus, we suggest that (1) LHb integrates mild stress experience, independently of pathological behavioural adaptations, and (2) alteration of GABA LTD may represent a threshold mechanism for the neural representation of mild stress accumulation. What is the functional relevance of keeping a synaptic trace of a mild stress exposure? It is possible that alteration of GABA LTD primes LHb neurons and facilitates further modulation by subsequent stressful experience. Indeed, stress models highlight the additive effects of intensity and recurrence of stressors over time, although stress levels recess during recovery phases (Savić et al., 2000). Such integration of stress history likely involves synaptic adaptations, which may include GABA LTD in the LHb. This is reminiscent of cumulative effects of addictive drugs on synaptic transmission in the mesolimbic reward system, where repetitive drug intake is necessary for pathological behaviour to emerge (Lüscher et al., 2020).

Altogether, our results identify a novel form of NO‐mediated LTD selective for inhibitory transmission in the LHb, with a low sensitivity threshold for stress‐induced perturbation. Thus, limiting GABA plasticity in the LHb may represent an eligibility trace for the cumulative integration of stress exposure.

## CONFLICT OF INTEREST

The authors declare no competing financial interests.

## AUTHOR CONTRIBUTIONS

All: Conceptualization, investigation, formal analysis and writing. ANP and AT: Investigation. MM: Conceptualization, investigation, formal analysis, writing and supervision.

### PEER REVIEW

The peer review history for this article is available at https://publons.com/publon/10.1111/ejn.15581.

## Data Availability

Data are available upon request to authors and as well deposited in Institutional repositories.
